# Emerging Therapeutics to Overcome Chemoresistance in Epithelial Ovarian Cancer: A Mini-Review

**DOI:** 10.3390/ijms18102171

**Published:** 2017-10-18

**Authors:** Robert Cornelison, Danielle C. Llaneza, Charles N. Landen

**Affiliations:** Department of Obstetrics and Gynecology, University of Virginia, Charlottesville, VA 22908, USA; jrc3hg@virginia.edu (R.C.); dcl5z@virginia.edu (D.C.L.)

**Keywords:** high-grade serous ovarian cancer, chemoresistance, multidrug resistance protein 1 (MDR1), epithelial–mesenchymal transition, DNA damage and repair, ribosome biogenesis

## Abstract

Ovarian cancer is the fifth leading cause of cancer death among women and the most lethal gynecologic malignancy. One of the leading causes of death in high-grade serous ovarian cancer (HGSOC) is chemoresistant disease, which may present as intrinsic or acquired resistance to therapies. Here we discuss some of the known molecular mechanisms of chemoresistance that have been exhaustively investigated in chemoresistant ovarian cancer, including drug efflux pump multidrug resistance protein 1 (MDR1), the epithelial–mesenchymal transition, DNA damage and repair capacity. We also discuss novel therapeutics that may address some of the challenges in bringing approaches that target chemoresistant processes from bench to bedside. Some of these new therapies include novel drug delivery systems, targets that may halt adaptive changes in the tumor, exploitation of tumor mutations that leave cancer cells vulnerable to irreversible damage, and novel drugs that target ribosomal biogenesis, a process that may be uniquely different in cancer versus non-cancerous cells. Each of these approaches, or a combination of them, may provide a greater number of positive outcomes for a broader population of HGSOC patients.

## 1. Introduction

Ovarian cancer remains a devastating diagnosis with an overall survival rate of ~40%, making it the fifth leading cause of cancer death in women and the most lethal gynecologic malignancy [1,2,3]. Over 220,000 women worldwide are diagnosed each year and an estimated 14,000 will die annually in the U.S. alone, a number that has only changed slightly after 30 years of research [4,5,6]. Histologically ovarian cancer is roughly composed of epithelial, germ cell and stromal tumors with epithelial ovarian cancer (EOC) being the most common and the most deadly. EOC can develop as high-grade serous, low-grade serous, endometrioid, clear cell and mucinous histotypes. It is now becoming clear that most serous cancers likely originate in the fallopian tube, although currently they are still referred to as ovarian cancers [7]. A more important distinction is high-grade versus low-grade cytologic subtypes, as low-grade serous ovarian cancers are more slow-growing, but more chemoresistant, than high-grade serous ovarian cancers (HGSOC). This review of chemoresistance will focus on HGSOC as it is the dominant subtype seen in the clinic. Importantly, until recently most preclinical studies have been performed on cell lines that are more of an endometrioid subtype than serous, and therefore may not be applicable to the tumor protein 53 (TP53)-mutant driven serous subtype [8].

First-line therapeutic interventions in ovarian cancer have evolved over the last few decades from a single nitrogen mustard alkylating agent to the current standard of care: cytoreductive surgery followed by combination taxane-platinum treatment [1,4,5]. About 60–80% of EOC patients receiving this combination after surgery will achieve complete remission, with ~80% of these having a chemoresistant recurrence [3,6,9]. Acquired platinum resistance remains a largely incurable condition and novel targeted therapeutics, new combination therapies, or innovative therapeutic strategies to specifically address the chemoresistant phenotype, are desperately needed [10,11,12]. Studies have elucidated many of the mechanisms that underlie the development of chemotherapy resistance in HGSOC (for reviews see [13,14,15,16,17]) and successfully targeting these systems in the clinic is critical in extending patient survival. This review will focus on HGSOC chemoresistance and emerging therapies that may show promise in mitigating and possibly defeating it.

## 2. Chemoresistance

Historically, the first major studies that encountered acquired resistance to therapy were the clinical trials in 1965 in pediatric hematopoietic malignancies by Frie et al. [18]. In using combinations of several cytotoxic agents, they saw the first real progress in extending the lives of children with leukemia. This success was cut short by recurrences where the leukemic cells had acquired the ability to resist treatment by hiding in a reservoir on the other side of the blood-brain barrier. The therapeutic agents used were unable to efficiently pass through the blood-brain barrier, leading to the patients eventually succumbing to multi-drug resistant disease. This gave the first clear evidence of dormant cancers taking advantage of our own defenses to resist treatment [19].

Broadly speaking, resistance to therapies is categorized into intrinsic or acquired resistance, although distinguishing between these two mechanisms can be difficult. Intrinsic resistance is the innate ability of the cancer cells to maintain and persist through their first exposure to treatment. Acquired resistance is the evolution of cancer cells, following treatment exposure, to an unaffected and persistent state whereby cells maintain and expand in the presence of subsequent therapies [13,20,21]. Acquired resistance can simply be thought of as microevolution: any survival advantage, whether geographic or molecular, will be clonally selected [22,23]. In terms of bacteria, this has been seen since the invention of antibiotics. In cancer cells, the overall story is similar but more complicated. One current hypothesis for chemoresistance is that a small percentage of a tumor consists of cancer stem cells or tumor initiating cells that are capable of self-renewal and recreating the full repertoire of cancer cells of the parental tumor as well as the expression of a distinctive set of surface biomarkers [13]. Terminology can vary here, as some would characterize this process as intrinsic resistance being present in a small population of cells. The presence of these cells creates unique challenges in addressing intrinsic and acquired resistance in chemoresistant tumors. For the purpose of this review, we will consider intrinsic resistance as being present when a patient progresses or incompletely responds to treatment, and acquired resistance when a patient achieves remission and later has a recurrence with resistant disease.

## 3. Intrinsic Versus Acquired Resistance

**Figure 1:**
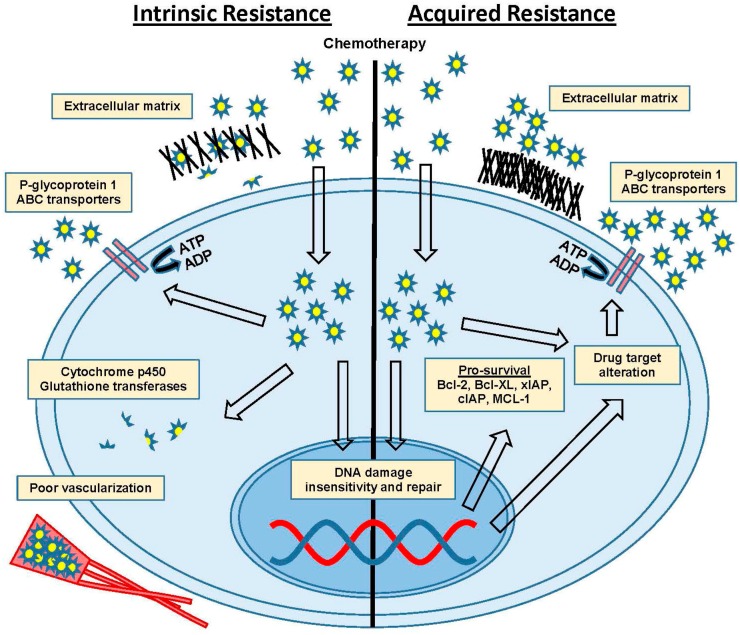
Intrinsic versus acquired resistance. Mechanisms of intrinsic and/or acquired chemoresistance include extracellular matrix proteins, P-glycoprotein (P-gp) efflux pumps and ATP binding cassette (ABC) transporters, cytochrome p450 and glutathione transferases, poor vascularization, DNA damage insensitivity and repair, drug target alteration and upregulation of pro-survival anti-apoptotic factors. Abbreviations: adenosine diphosphate (ADP), adenosine triphosphate (ATP), B-cell lymphoma 2 (BCL-2), B-cell lymphoma-extra large (BCL-XL), myeloid cell leukemia 1 (MCL-1), X-linked inhibitor of apoptosis protein/cellular inhibitor of apoptosis (xIAP/cIAP).

The inherent ability of cells to survive chemotherapy can be mediated through several distinct mechanisms. Drug efflux pumps (adenosine triphosphate (ATP) binding cassette (ABC) transporters) lower drug concentrations within cells (reviewed in [24] with follow-up [25]), and therapeutic agents can be biochemically degraded through detoxifying enzymes such as cytochrome p450 and glutathione transferases (reviewed in [26,27]). Poor vascularization can lead to decreased drug concentration at the tumor site (reviewed in [28,29]), and extracellular matrix (ECM) interactions and secreted factors can influence tumor environment mediated resistance (reviewed in [30]). These intrinsic mechanisms of resistance are critical in determining initial response to therapies and may influence subsequent outcomes that lead to acquired resistance (Figure 1).

Acquired resistance is developed by the step-wise, molecular evolution of tumor cells through natural selection of changes giving a survival advantage [31]. This can include modulation of the expression of genes that drive increased anti-apoptotic signaling (i.e., X-linked inhibitor of apoptosis protein/cellular inhibitor of apoptosis protein (xIAP/cIAP), B-cell lymphoma 2 (BCL-2), B-cell lymphoma-extra-large (BCL-XL), and myeloid cell leukemia 1 (MCL-1)), DNA repair capacity, and the tolerability of genetic damage. Other alterations can be decreased sensitivity to DNA damage checkpoint signaling, or changes to ECM-collagen VI surface proteins, and the genes responsible for interacting with the ECM and stromal cells surrounding the tumor [4,11,15,32,33,34,35]. Cells can also acquire changes to drug targets that are mediated through multiple mechanisms [36,37], or in the case of some targeted therapies, by simply bypassing the target through an alternate pathway [38,39]. The simplest example of this is seen in androgen therapy for prostate cancer. After androgen deprivation therapy, the cells amplify the level of androgen receptor to the point that the levels are too high to stoichiometrically defeat [40]. Thus, acquired resistance can dramatically alter tumor cells to a point that they can be resistant to one or several types of therapy, but may also become uniquely sensitive to other therapies that have no effect on treatment-naïve cancer cells.

Many cancers, particularly HGSOC, are characterized by marked intratumoral heterogeneity. Overall, cancer is a disease of heterogeneity. The inherent genetic instability that gave rise to the tumor cells gives each the ability to quickly respond to changes in its local molecular microenvironment. This gives tumors the ability to use multiple combinations of these adaptive intrinsic and acquired mechanisms of resistance to defeat most modern chemotherapies and targeted therapeutics. For HGSOC, studies have focused on drug efflux and enhanced DNA damage repair capacity as being the critical, and druggable, mediators of resistance.

## 4. Efflux Pumps and MDR1

**Figure 2:**
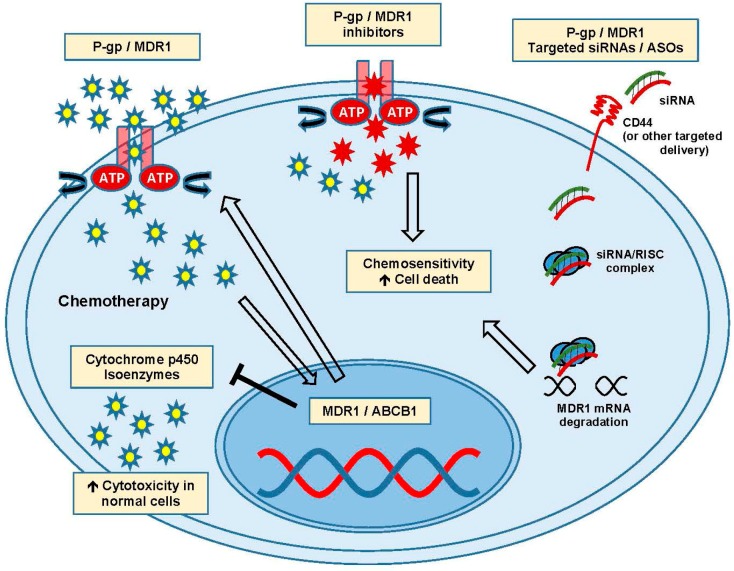
Action and targeting of P-gp and MDR1 drug efflux pumps. Activation of MDR1/ABCB1 transcription leads to drug efflux and inhibition of cytochrome p450 isoenzymes. Chemosensitivity and cell death can be enhanced by inhibitors and siRNA targeted therapies. Abbreviations: adenosine triphosphate binding cassette-B1 (ABCB1) transporters; antisense oligonucleotide (ASO), cluster of differentiation 44 (CD44), multidrug resistance protein 1 (MDR1), small interfering RNA (siRNA).

The ability of cancer cells to lower the intracellular concentration of drugs is well documented and the ATP binding cassette (ABC) transporters have been extensively studied in cancer chemoresistance. They are a diverse group of ATP-dependent efflux pumps containing 48 different family members with a common structural theme: a variable transmembrane domain and a conserved nucleotide binding domain [32,41]. In action, the transmembrane domain binds its target which induces a conformation change, removing the bound substrate from the cell using the hydrolysis of ATP for energy [24,25,42]. In ovarian cancer chemoresistance, the most commonly targeted of these transporters are the ABCB subfamily, with multidrug resistance protein 1 (MDR1, also known as P-glycoprotein (P-gp) and ABCB1) being the most exhaustively studied (Figure 2). The other ABC transporters appear to be limited in their ability to use paclitaxel as a substrate, with MDR1 being the primary P-gp responsible for reducing its intracellular concentrations [5,37,43].

MDR1 is responsible for the efflux of a truly staggering number of toxic compounds with diverse properties [44]. Several large studies have been undertaken and identified single nucleotide polymorphisms in MDR1 that are predictive of response to therapy, [45,46] with several promising drugs emerging to reduce their effects [42,47]. Over the last 15 years, intense research has explored strategies to clinically inhibit MDR1 with small molecules. Resistance to taxane therapy in ovarian cancer appears to occur through multiple mechanisms depending on the genetic background of the cells, but MDR1 efflux is thought to be the primary mechanism [20,39]. Eliminating MDR1-mediated paclitaxel/cisplatin efflux specifically has been shown to reverse resistance in the laboratory, but translating this promising outcome to the clinic has been incredibly challenging [48,49,50]. Drugs that inhibit MDR1 can also inhibit some cytochrome p450 isoenzymes, which are necessary to detoxify non-cancerous cells of the cytotoxic chemotherapies typically used. Targeting proteins responsible for protecting cells from toxic molecules is problematic, and combining MDR1 inhibition with cytotoxic chemotherapy can cause unpredictable and dangerous side effects [51,52].

First generation small molecule inhibitors of MDR pumps have had limited success due to these unforeseen consequences and overall toxicity problems, with clinical trials being marred by poor tolerability [53,54,55]. Second generation inhibitors with minimal cytochrome p450 inhibition showed promise but generally targeted multiple ABC transporters, resulting in negative effects in a diverse set of tissues. Using this information, combined with advances in structural chemistry and drug design, third generation P-gp inhibitors appear to be more specific and better tolerated, with several currently being clinically investigated [56,57]. Apatinib has been found to reverse paclitaxel resistance in both in vitro and in vivo patient-derived xenograft (PDX) model systems [58]. These third generation agents, as well as Tariquidar, have shown significant improvement in potency, specificity and tolerability with clinical trials ongoing [59]. Ovarian cancer groups are also investigating these latest agents using novel encapsulation methods to deliver them preferentially to tumor cells, with some success [60]. Overall pharmacologic inhibition of the efflux pumps remains challenging due to limits in balancing toxicity risks against therapeutic gains.

Targeting the MDR1 mRNA for degradation with small interfering RNA/antisense oligonucleotide (siRNA/ASO) is also being investigated as a method to ensure target specificity, and combining this with nanoliposomes that target cancer cells has been attempted in an effort to limit toxicity [47,54]. Yang et al. published findings on self-assembling nanoparticles that target cluster of differentiation 44 (CD44) to deliver MDR1 siRNA specifically to cancer cells in a mouse PDX model of ovarian cancer. When used in combination with paclitaxel, the siRNA delivery was specific to the cancer cells as increased sensitivity to paclitaxel was demonstrated [61]. While these modifications in drug delivery and specificity may alleviate some toxicity hurdles observed in the treatment of chemoresistant disease, it is also important to consider therapies that do not directly overlap with primary mechanisms of chemoresistance.

The development of novel tubule stabilizers that are not substrates for MDR1 efflux, such as the epothilone family, has shown efficacy in cells and PDX models previously resistant to paclitaxel [62,63,64,65]. Ixabepilone has been shown to have inhibitory concentrations of 50% (IC_50_s) an order of magnitude lower than typical concentrations seen with paclitaxel, and also has less neuropathies and other side effects in patients [62]. Positive clinical outcomes have been seen using Ixabepilone in recurrent ovarian cancer in combination with bevacizumab, and studies are ongoing to determine optimum patient enrollment criteria [66]. These results may highlight the utility of combination therapies that are not directly targeting major mechanisms of chemoresistance in ovarian cancer [35].

While some success has been observed in targeting MDR1 to overcome resistance, any real clinical gain has remained elusive. A recent whole genome sequencing study of a cohort of chemoresistant ovarian cancer has shown MDR1 to be rearranged in some chemoresistant populations, with promoter fusion driving upregulation. The promiscuous nature of the ABC transporters combined with the diverse side effects and toxicity problems has prompted some investigators to call for a change in tactics. Moving upstream of MDR1 to the epithelial–mesenchymal transition (EMT) pathway ties together multiple mechanisms associated with the acquisition of resistance, and EMT has a special position in ovarian cancer progression.

## 5. Epithelial–Mesenchymal Transition

**Figure 3:**
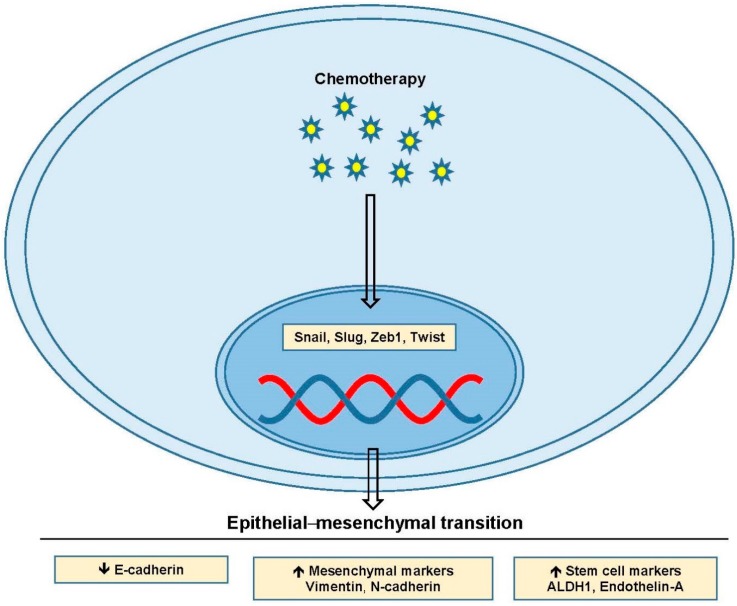
Epithelial–mesenchymal transition. Upregulation of Snail, Slug, Zeb1 and Twist lead to changes in mesenchymal and stem cell markers including downregulation of E-cadherin, and upregulation of vimentin, N-cadherin, ALDH1 and endothelin-A. Abbreviations: aldehyde dehydrogenase-1 (ALDH1).

EMT is the process of epithelial cells losing their apical-basal polarization, detaching through loss of adhesion, and becoming mesenchymal-like in both appearance and invasiveness [67,68]. There are myriad genetic changes involved in the process with cells typically losing expression of the adhesion protein E-cadherin, along with several of the tight junction proteins [67,69]. During EMT, cells begin expressing mesenchymal markers like vimentin and N-cadherin, as well as markers associated with stem cell populations such as aldehyde dehydrogenase-1 (ALDH1) and endothelin-A [13,70,71]. These changes are driven by the transcription factors Snail, Slug, Zeb1 and Twist, and many of the key pathways involved in ovarian cancer regulate their expression (Figure 3) [72,73,74,75]. EMT is seen after chemotherapy in surviving ovarian cancer cells and it’s been shown that Snail and Slug EMT transcription factors are upregulated [68,74,76,77]. Thus, factors altered in EMT are frequently overexpressed in ovarian cancer and are likely contributory to chemoresistance.

Chemoresistant phenotypic changes in ovarian cancer appear strongly correlated with EMT and the presence of subpopulations of cancer stem cells [10,68,71,74,78]. The pro-survival, anti-apoptotic signaling, efflux pump overexpression, and resistance to DNA damage seen in chemoresistant populations can all come from sustained EMT [69,75,79]. One relatively unique property of ovarian cancer is its fondness for metastasizing to the omentum, and interactions with this fatty compartment may play a critical role in the development of chemoresistance [3,80]. HGSOC does not require a hematologic route of metastasis as the entire peritoneal cavity is accessible from its site of origin, and HGSOC appears to readily shed and establish colonies throughout the chest cavity [80]. This shedding has been shown to involve EMT and the formation of tumor spheroids, which are protected by cancer-associated fibroblasts (CAFs) and hijacked immune system effector cells [3]. Ovarian cancer has been shown to be capable of responding to chemotherapy, with an acquired EMT phenotype giving rise to chemoresistance [67,81]. The endothelin receptor has been identified as a major source of EMT changes in response to chemotherapy, and blocking it has shown some reversal of chemoresistance [68]. Zibotentan is an antagonist of endothelin receptor A (ETA) [82]. After failing initial phase III trials, this drug is still being investigated, and its utility in chemoresistant disease in ovarian cancer is still promising. Targeting EMT in ovarian cancer may both stop the acquisition of chemoresistance and inhibit tumor progression and metastasis; however, cells that continue to evade death may have greater abilities to endure in the face of severe DNA damage.

## 6. DNA Damage Tolerance and Repair Capacity

DNA damage response pathways are very well characterized and generally signal through checkpoint kinase 1 and checkpoint kinase 2 (CHK1/CHK2) to determine the amount of damage present, amplify the repair signal to enhance repair efficiency, and delay the cell cycle to allow time for repair [83]. Damage is detected by the MRE11/RAD50/NBS1 (MRN complex), which signals through the phosphotidylinositol 3-kinase (PI3) family members ataxia telangiectasia mutated (ATM), Rad-3-related (ATR) and DNA-dependent protein kinase (DNA-pk) by phosphorylation of H2A histone family member X (H2AX). This in turn amplifies the repair signal, and enhances the buildup of DNA repair pathway members at the site of damage [84,85]. In order to allow sufficient time for repair, the cell cycle must be halted. The Gap2-Mitosis (G2-M) boundary is maintained through phosphorylation of cell-division cycle 2 (CDC2) on threonine 14 and tyrosine 15 by WEE1-like (WEE1) and myelin transcription factor 1 (MYT1) kinases, which sequester the CDC2/cyclin B complex in the cytoplasm [86,87,88,89]. For mitosis to proceed, CDC2 must be activated by cell-division cycle 25 (CDC25) dephosphorylation and translocation to the nucleus [90,91]. The delay is induced by ATM and ATR activation of the CHK2/CHK1 kinases, respectively. CHK2/CHK1 phosphorylate CDC25B, inhibiting its phosphatase activity on CDC2 and leaving the inactive complex in the cytoplasm [83]. Thus, the cell cycle is tightly regulated by several mechanisms, and sensitivity or resistance to treatments may involve alterations in these processes that promote imminent death or allow for recovery and repair.

Platinums are the drug of choice for treating many aggressive cancers, and most HGSOC are quite sensitive to its toxicity [92]. The mechanisms of platinum toxicity have yet to be fully elucidated, but it’s thought to act as a severe DNA damaging agent, creating inter- and intra-strand cross links from the formation of platinum:DNA adducts [93]. Ovarian cancers presenting with some form of homologous recombination (HR) deficiency generally respond better to DNA damaging therapies like cisplatin, and also may benefit from poly(ADP-ribose) polymerase-I (PARP I) inhibitor therapy [94,95]. PARP inhibitors exploit the broken DNA repair pathway to act as a synthetic lethal [96]. The deficient repair system also increases the efficacy of DNA damaging agents, depending on the specific gene mutation, and improves overall survival. Ovarian cancers where there are no HR deficiencies are less well-defined and may represent an early step in tumor initiation or chemoresistance [95].

**Figure 4:**
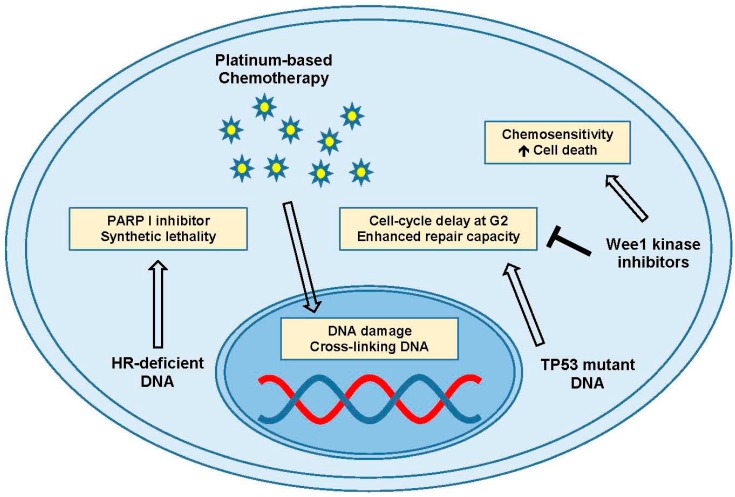
DNA damage and repair. PARP inhibitors in the presence of platinum-based chemotherapy and homologous recombination (HR) deficient DNA can induce synthetic lethality. TP53 mutated DNA can show cell cycle delay at the G2 phase, which can be inhibited by Wee1 kinase inhibitors to promote cell death and enhance chemosensitivity. Abbreviations: Gap 2 (G2); poly(ADP-ribose) polymerase-I (PARP I); tumor protein 53 (TP53).

TP53 mutant cells are thought to have a deficient G1 arrest ability with an enhanced G2 delay, allowing them to cope with genetic damage [97,98]. Cancer cells over time can become resistant to platinum-induced DNA damage and apoptosis through G2 delay and enhanced repair (Figure 4) [99,100]. The HGSOC combination of TP53 loss and HR repair deficiency has led many groups to ask the question: can we force cells with severe DNA damage through the cell cycle by attacking the delay mechanisms, and lead cells to death by apoptosis or mitotic catastrophe? CDC2 phosphorylation by Wee1 kinase is an attractive drug target for this question with Wee1 kinase specific inhibitors, such as MK-1775 (low nanomolar IC_50_), showing promising results [101,102]. PD0166285 is a newer Wee1 inhibitor and G2 checkpoint abrogator [103,104]. With HGSOC being almost entirely TP53 mutant, removing the G2 arrest has been shown to reverse platinum resistance, and targeting this may help alleviate chemoresistance in some subpopulations of ovarian cancer [105]. Other mechanisms of chemoresistance that may be required for the upregulation of any and all processes related to DNA damage repair, EMT, drug efflux, and the capacity of the cell to utilize intrinsic mechanisms of resistance, and develop acquired mechanisms of resistance, are currently under investigation as potential targeted therapies for HGSOC.

## 7. Angiogenesis Inhibitors

Angiogenesis has long been a hallmark of most malignancies and it is well characterized in HGSOC. Anti-angiogenic therapies for HGSOC have been FDA approved for use in the recurrent setting, and while showing an increase in progression free survival (PFS) there has been no change to overall survival (OS) [106,107]. Targeting vascular endothelial growth factor (VEGF) and VEGF receptor (VEGFR) in HGSOC showed only transient benefit with resistant disease occurring in many cases (reviewed in [108,109]). Alternative pathways were identified that cancer cells could use to bypass the VEGF blockade with angiopoietins being shown to play a critical role in mediating neovascularization and response to antiangiogenic therapies [110,111].

Recent phase III clinical trials in HGSOC have shown a significant increase in PFS with the same lack of OS increase in platinum resistant HGSOC with both bevacizumab and trebananib [112,113]. While the early excitement of angiogenesis inhibitors has diminished, the increase in PFS is not an insignificant endpoint in HGSOC. The continued investigation of combinations of angiogenesis inhibitors and identification of biomarkers for better screening criteria may still show clinical benefit.

## 8. PARP Inhibitors

Using poly(ADP-ribose) polymerase (PARP) inhibitors for treating breast cancer 1 (BRCA) or HR deficient HGSOC is a highly active area of research with several FDA-approved therapies in the recurrent and maintenance setting [114]. PARP is an enzyme responsible for the correct repair of DNA damage, and it does this through directly interacting with the repair machinery on the damaged site. PARP has a strong affinity for single strand DNA breaks and autoactivates, forming chains of PAR attached to the DNA adjacent to the break. These polymers act as a scaffolding system to recruit and assemble the various repair system effector molecules [115]. If PARP is functionally inhibited these single strand DNA breaks with attached PARP polymers will evolve into double strand breaks (DSBs) due to replication fork collision [116]. In cancer cells with deficient HR repair systems these DSB’s will build up over time whereas normal cells have the capacity to repair them [117]. By using small molecules to ‘trap’ PARP on the DNA, cancers deficient in DNA repair will be preferentially targeted in a synthetic lethal fashion as a result of being unable to clear the polymer chains [118,119,120].

Resistance to PARP inhibitors develops rapidly in some patients and occurs in surprising ways (reviewed in [121]). Reversion of mutated DNA repair genes back to functionally normal, full length forms being one method commonly seen [122,123,124]. Other mechanisms alter the complex balance between non-homologous end joining and HR repair through microRNAs (miRNAs) [125], epigenetics [126] or nascent, truncated BRCA mutants [127,128]. Upregulation of the MDR1 efflux pumps has also been shown to induce resistance to PARP inhibitors [129] with drugs identified that can specifically reverse this [130].

## 9. Exosomes and Cancer

Exosomes are extracellular vesicles <100 nm in diameter that were initially thought of as cellular waste disposal units [131]. Research has shown a diverse array of functions for exosomes (reviewed in [132]) and they generally act as a form of an intracellular communication system [133]. Cancer cells are generally seen as releasing more exosomes than normal cells and evidence is building that exosomes play a crucial role in cancer progression, metastasis, and chemoresistance [134,135,136,137].

Exosomes have been found to play a role in resistance to chemotherapy [138], and in HGSOC have been shown to transmit platinum resistance to sensitive cells through intracellular communication of EMT signals [139]. Cao et al. showed exosomes mediating platinum resistance through DNA methyltransferase I (DNMT1) [140]. DNMT1 is a regulator of a large array of developmental processes and plays a critical role in regulating DNA damage response and repair [141]. If exosomes are capable of controlling such critical aspects of cellular responses to chemotherapy, then exosomal inhibitors may be used as a targeted therapeutic [134,135].

The idea that exosomes are also capable of sending out tumor specific signals begs the question: can we use exosomes as a cancer specific targeting system for drug delivery? Evidence is building for this being a promising way to deliver toxic therapies directly to the tumor [142] with Hadla et al. illustrating their use in ovarian cancer [143].

## 10. Checkpoint Inhibitors

The most commonly-used therapeutic strategy in HGSOC today is combined platinum/taxane treatment after surgery. Several other options are becoming more clinically viable. The discovery of immune system checkpoint inhibition, like programmed cell death-1 (PD-1), has revolutionized the treatment of several types of cancers and is being actively explored in HGSOC [144,145].

The immune system has a dedicated surveillance network for identifying and destroying cells displaying neoplastic changes, neoantigens or other signals of dysfunction. Tumors, as they form, generally set off various checkpoint alarms and inflammatory processes that signal to the immune effector cells. For any growing lesion to form a viable tumor it has to evade immune system detection. PD-1 is one of a host of cell surface receptors used to identify cells to the immune system as “self” to stop normal tissues from being attacked. Many types of tumors have used this as a mechanism to hide from immune system detection, and blocking this escape tactic has shown efficacy in a diverse set of cancers. In HGSOC, trials have shown positive results using both anti-PD-1 antibody therapies as well as antibodies directed at programmed death-ligand 1 (PD-L1), its normal ligand [146,147]. HGSOC has been identified as having high expression of the PD-1 receptor and PD-1 inhibitors such as pembrolizumab and nivolumab are in FDA trials [146]. Cytotoxic T-lymphocyte associated protein 4 (CTLA-4) is another checkpoint inhibitor that is in trials and ipilumab is currently under investigation in HGSOC [148].

Resistance to checkpoint inhibitors is both intrinsic and acquired, with intense research underway determining sensitivities in many tumor types. The recognition system appears to have multiple compensatory factors interacting, and blockade of a single receptor has been shown to compensate with upregulation of parallel pathways [145]. Several examples have been identified of tumors making dramatic changes to their cell surface markers to evade the immune system entirely. Deletion of major histocompatibility complex (MHC) class II has been seen and presents a difficult resistance mechanism to overcome [149]. However, as broad use of checkpoint inhibitors is relatively recent, more information on resistance mechanisms is anticipated.

## 11. Ribosomal Biogenesis

One of the first identified hallmarks of cancer, dating back to the late 1800’s, was enlarged and/or pronounced nucleoli [150]. The size of the nucleoli is generally thought to reflect the amount of ribosomal biogenesis happening in the nucleolus, and proliferating cells typically require a large amount of ribosomes to progress through the cell cycle [151]. Ribosomes are critical molecular machines common to all eukaryotic cells that allow the conversion of RNA messages to nascent proteins. Ribosomal synthesis and processing is one of the most energetically demanding activities cells undergo, and consequently their synthesis is also one of the most regulated systems in eukaryotic life [152]. Altering the rate of ribosomal biogenesis can lead to TP53 stabilization, representing a phenotype known as nucleolar stress [153,154,155]. Given that ribosomal biogenesis is central to life and appears to play a prominent role in cancer, it’s important to consider if and how this system can be targeted in cancer cells specifically.

Recently the idea of targeting ribosomal biogenesis has come forward as a viable strategy for treating various forms of cancer [156,157] and specifically for HGSOC [158]. Ribosomal synthesis begins with the formation of the RNA polymerase I (RNA Pol I) pre-initiation complex, followed by transcription of the 47 s pre-ribosomal RNA. The pre-initiation complex is set up first by binding of the high mobility group protein upstream binding transcription factor (UBTF) within the ribosomal DNA (rDNA) promoter region, ejecting the H1 histone. This interaction is then stabilized by the binding of the selectivity complex selective factor 1 (SL1), which commits the promoter for transcription by multiple rounds of RNA polymerase I. After transcription, a complex series of modifications spanning several subcellular compartments leads to the assembly of functional ribosomes [159,160]. Although there are several key players in ribosomal biogenesis, there may be mechanisms that are specific and can be targeted in cancer cells without negatively impacting the majority of normal cells.

**Figure 5:**
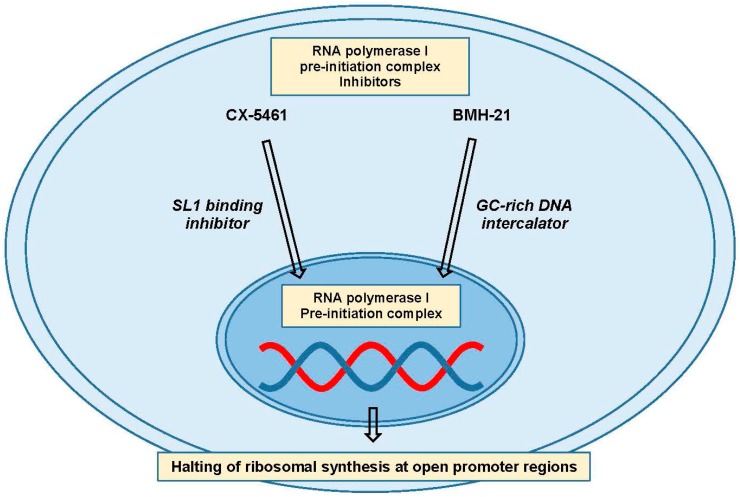
Ribosomal biogenesis targeting. Inhibitors of the RNA polymerase I pre-initiation complex, such as CX-5461 and BMH-21, can inhibit ribosome production and availability. Some data suggests that cancer cells may be more susceptible to targeting this pathway than nontransformed cells. Abbreviations: selective factor 1 (SL1).

RNA polymerase I is only responsible for the transcription of the pre-ribosomal RNA, so targeting it with small molecule inhibitors appears to be the most appealing strategy for specifically shutting down ribosomal synthesis [156,161]. Two specific pol I inhibitors, CX-5461 and BMH-21, have been developed, and have slightly different substrates (Figure 5). CX-5461 is thought to inhibit the binding of SL1 to the rDNA promoter, leading to pre-initiation complex failure and possibly an abnormal chromatin structure picked up as a form of damage [162,163,164]. BMH-21, on the other hand, is a GC-rich DNA intercalating agent that inhibits formation of the pre-initiation complex leading to RNA polymerase I subunit A (RPA194) degradation, the major subunit of the RNA pol I holoenzyme [165,166]. These inhibitors may begin to shed light on the possibility and efficacy of targeting ribosomal biogenesis in cancer, and differences in their activity may shed light on the most crucial aspects of RNA Pol I inhibition.

## 12. Summary

In summary, while the understanding of mechanisms underlying intrinsic and acquired chemoresistance in ovarian cancer is expanding rapidly, overall therapeutic options remain limited. Promising results generated in vitro showing the therapeutic potential of targeting MDR1 have fallen short in practical application in the clinic due to toxicity. Alterations in EMT targets have had minimal success given there are several mechanisms remaining active that allow the tumor to adapt. As well, agents that try to exploit high levels of DNA damage may only be clinically applicable in contexts where tumors are also HR deficient.

The pitfalls and shortcomings of translating these novel targets and associated drugs to the clinic further highlight the essential need for approaches to treatment that go beyond targeting single mechanisms. Mechanisms targeted previously only represent a handful of players in a complex and heterogeneous system that comprises chemoresistance in ovarian cancer. With all the new information on mechanisms of resistance to cytotoxic agents, specific resistance mechanisms to emerging targeted therapies and whole genome characterization of HGSOC personalized medicine may be on the horizon, and required for durable cures. Molecular characterization of the tumor followed by prediction of optimal therapeutic combinations with inhibitors blocking the expected paths of resistance may be the only way to achieve an increase in overall cure rates. The novel approach of targeting ribosomal biogenesis may prove more useful than previous approaches in improving outcomes for a larger proportion of patients with chemoresistant ovarian cancer in the future; however, previous research provides hope that it is feasible to identify the most important mediators of chemoresistance, while exploring the therapeutic potentials of current novel targets, so a broader population of patients with chemoresistant ovarian cancer will experience positive outcomes. As the understanding of resistance with tumor heterogeneity is enhanced, it is hopeful that targeting the specific chemoresistant population with upfront or consolidation therapy will lead to absolute cures, rather than just modest responses to therapy.
